# Detecting common coccinellids found in sorghum using deep learning models

**DOI:** 10.1038/s41598-023-36738-5

**Published:** 2023-06-16

**Authors:** Chaoxin Wang, Ivan Grijalva, Doina Caragea, Brian McCornack

**Affiliations:** 1 Department of Computer Science, Kansas State University, Manhattan, KS 66506 USA; 2 Department of Entomology, Kansas State University, Manhattan, KS 66506 USA

**Keywords:** Image processing, Machine learning, Entomology, Agroecology

## Abstract

Increased global production of sorghum has the potential to meet many of the demands of a growing human population. Developing automation technologies for field scouting is crucial for long-term and low-cost production. Since 2013, sugarcane aphid (SCA) *Melanaphis sacchari* (Zehntner) has become an important economic pest causing significant yield loss across the sorghum production region in the United States. Adequate management of SCA depends on costly field scouting to determine pest presence and economic threshold levels to spray insecticides. However, with the impact of insecticides on natural enemies, there is an urgent need to develop automated-detection technologies for their conservation. Natural enemies play a crucial role in the management of SCA populations. These insects, primary coccinellids, prey on SCA and help to reduce unnecessary insecticide applications. Although these insects help regulate SCA populations, the detection and classification of these insects is time-consuming and inefficient in lower value crops like sorghum during field scouting. Advanced deep learning software provides a means to perform laborious automatic agricultural tasks, including detection and classification of insects. However, deep learning models for coccinellids in sorghum have not been developed. Therefore, our objective was to develop and train machine learning models to detect coccinellids commonly found in sorghum and classify them according to their genera, species, and subfamily level. We trained a two-stage object detection model, specifically, Faster Region-based Convolutional Neural Network (Faster R-CNN) with the Feature Pyramid Network (FPN) and also one-stage detection models in the YOLO (You Only Look Once) family (YOLOv5 and YOLOv7) to detect and classify seven coccinellids commonly found in sorghum (i.e., *Coccinella septempunctata*, *Coleomegilla maculata*, *Cycloneda sanguinea*, *Harmonia axyridis*, *Hippodamia convergens*, *Olla v-nigrum*, Scymninae). We used images extracted from the iNaturalist project to perform training and evaluation of the Faster R-CNN-FPN and YOLOv5 and YOLOv7 models. iNaturalist is an imagery web server used to publish citizen’s observations of images pertaining to living organisms. Experimental evaluation using standard object detection metrics, such as average precision (*AP*), *AP*@0.50, etc., has shown that the YOLOv7 model performs the best on the coccinellid images with an *AP*@0.50 as high as 97.3, and *AP* as high as 74.6. Our research contributes automated deep learning software to the area of integrated pest management, making it easier to detect natural enemies in sorghum.

## Introduction

Pest management is a strategy used to control any living organism that poses a risk to our food, fiber, and health security. It has played an essential role in achieving the current food supply, and its role will continue to be critical in any agricultural production system^1^. Since the beginning of agricultural development, growers have had to compete with harmful insects, collectively called ‘pests’^2^. These organisms can reduce crop yields and fruit quality, damage plants, serve as disease vectors, and contaminate food crops. Different strategies have been developed to control arthropod pests in agriculture, including chemical, cultural, biological (e.g., plant resistance, natural enemies, etc.), and mechanical methods^3^. There is much concern about the use of chemicals for pest control due to their cumulative non-sustainable adverse effects on the environment^4–6^, particularly non-target effects on beneficial organisms, including natural enemies (e.g., predators, parasitoids, microorganisms) and pollinators^7,8^, and the potential for the development of pesticide resistance^9,10^.

A valuable tool for addressing this threat is integrated pest management (IPM). IPM was developed in the early 1970s as a pest control strategy that promotes sustainable agriculture with a strong ecological basis^11^. IPM is an approach that incorporates various tactics to control all classes of pests (e.g., insects, pathogens, weeds, vertebrates) to create an ecologically and economically efficient production system^11^. These tactics include biological control, cultural practices, host-plant resistance, genetic manipulation, and pesticides^3,12,13^. IPM tactics have been applied in different crops, including sorghum production. Sorghum [*Sorghum bicolor* (L.) Moench] is the fifth most valuable cereal crop globally^14^. In the U.S., this crop had a value of more than $1 billion and was planted on 5.26 million acres in 2019^15^. Sorghum production in the world is used mainly for human consumption and animal feed; in the U.S., it is used as livestock feed and turned into ethanol. However, the current production of sorghum faces significant pest management challenges. Since 2013, with the outbreak of *Melanaphis sacchari* (Zehntner) (Hemiptera: Aphididae), commonly named sugarcane aphid (SCA), different tactics have been developed, including scouting protocols, pesticides treatment guides, and host plant resistance programs to prevent yield losses in sorghum^16^.

Proper identification and classification of insect pests at an early stage are important tasks in crops because pest management strategies (i.e., pesticides and cultural control methods) can be costly and overused when misidentification happens. However, insect pests are not the only factor that affects our understanding of pest management in agriculture. Sorghum farmers encounter other beneficial insects that need to be identified and classified automatically to better understand the pest and beneficial insects interactions (i.e., predation) during pest scouting in fields. One of the major communities feeding on SCA are lady beetles (Coleoptera, Coccinellidae). Common genera, species, and subfamily levels of coccinellids that we can find on sorghum plants include *Coccinella septempunctata*, *Coleomegilla maculata*, *Cycloneda sanguinea*, *Harmonia axyridis*, *Hippodamia convergens*, *Olla v-nigrum*, and the subfamily Scymninae^17^. Technological advances in artificial intelligence and machine learning related to how living organisms can be most efficiently identified and classified with the smallest use of labor and time to increase precision agriculture represent a major focal point of modern agricultural production and research.

Machine learning is a sub-field of artificial intelligence, in which labeled data can be used to train a model, and the trained model can be subsequently used to make inferences and predictions on new incoming data without additional programmatic effort^18^. Convolutional neural networks (CNN)^19^ represent a type of machine learning model, more specifically, a deep learning model, which can be used to analyze visual imagery. CNNs excel at a variety of computer vision tasks, such as image classification, object detection and localization, among others. Object detection refers to the task of identifying and classifying instances of objects of interest in images or video frames^20^. CNN-based approaches for object detection extract features from the input image and use the features to perform two main tasks: 1) detect regions of interest (ROI) as bounding boxes that contain instances of objects in the image (a.k.a., object identification); and 2) classify ROIs into an arbitrary number of classes (a.k.a., object classification). Depending on how these two tasks are performed, object detection approaches can be classified as one-stage detectors and two-stage detectors^21^. One-stage detectors perform both tasks simultaneously in one stage, and include models in the YOLO family^22–28^, among others. Two-stage detectors identify ROIs in a first stage, and subsequently classify the ROIs and refine their bounding boxes in a second stage. Two-stage detectors include models such as Faster R-CNN^29^, Cascade R-CNN^30^ and FPN^31^. Traditionally, two-stage detectors have been more accurate than one-stage detectors, while the one-stage detectors have been faster and more suitable for use in practical applications that require real-time object detection^32^. Remarkably, combinations of the Faster R-CNN^29^ and FPN^31^ networks have achieved competitive results on popular benchmark datasets^21,33^. However, some of the recent YOLO models^27,28,34^ have produced state-of-the-art performance both in terms of accuracy and speed on benchmark datasets, with YOLOv7 being known to produce the best results at the end of year 2022^28^.

Deep learning software for object detection can be designed in a user-friendly manner and allows for the training of models that can be applied to solve agricultural challenges^18^. Recent studies using deep learning neural networks for object detection have shown that it is possible to develop models for automated disease identification and insect recognition^35–37^. Some studies have focused on the use of object detection approaches to identify and classify pests based on images of yellow sticky traps and other types of insect traps^38–47^. For example, Salamut et al.^40^ focused on detecting cherry fruit flies based on yellow sticky trap images. Several one-stage and two-stage object detection approaches were compared, including Faster R-CNN and YOLOv5^26^ using a dataset that contains 1,600 annotated images. The best results overall were obtained using a Faster R-CNN model with lightweight MobileNet^48^ as the backbone network. Specifically, the Faster R-CNN model had average precision *AP*@.50 of 0.88% as compared to the best YOLOv5 model, which had an *AP*@0.50 of 0.76%. Wang et al.^42^ published a dataset (called Pest24) of approximately 25,000 pest trap images that contain 24 field pests. They trained several object detection models on this dataset, including Faster R-CNN (with VGG-16 as the backbone network), Cascade R-CNN (with ResNet-50-FPN) and YOLOv3^24^ (whose backbone is called Darknet-53). Experimental results showed that YOLOv3 had the best performance on this dataset, with an overall mean average precision (*mAP*@0.50) of 59.79% as compared to an *mAP*@0.50 of 57.23% for Cascade R-CNN and an *mAP*@0.50 of 51.10% for Faster R-CNN. Li et al.^38^ used the Faster R-CNN model pre-trained on the COCO dataset^49^ to detect small pests (whitefly and thrips) using a dataset of approximately 1,500 sticky trap images and showed that the model transferred from COCO is more accurate than the corresponding model trained directly on pest images.

Wang et al.^50^ adapted the Faster R-CNN model to make it easier to find small pests in light-trap images. The improved model used the attention mechanism^51^ to focus on more predictive features, together with a sampling strategy for the region proposal network to address class imbalance and also an adaptive RoI selection to select best features from different levels of a pyramid network. Experimental results on a dataset (called AgriPest21) of approximately 25,000 images with 21 types of pests showed that the adapted model achieved a *mAP* of 78.7%, which was significantly better than the *mAP* of the baseline models included in the comparison study (both one-stage, e.g. SSD^52^ and two-stage models, e.g., Cascade R-CNN^30^). Jiao et al.^53^ also used an adaptive feature fusion pyramid network to identify richer features for pest detection together with Faster R-CNN network (with ResNet50 as backbone) and obtained a competitive *mAP* value of 77.4% on the AgriPest21 dataset^50^. Zhang et al.^44^ used strategies similar to those in^50,53^ (i.e., attention mechanism to obtained better features, fusing features from a pyramid network) to adapt YOLO models to small pest detection tasks. Experimental results on the Pest24 dataset^42^ showed that the adapted YOLO model (called AgriPest-YOLO) had better performance than Faster R-CNN, Cascade R-CNN and several YOLOv4^25^ and YOLOv5^26^ variants, producing an overall *mAP*@0.50 of 71.3% and *mAP*@0.50 : 0.5 : 0.95 of 46.9%.

As opposed to the abovementioned studies that focused on images of trapped pests, other studies have focused on pest detection in the wild^54–59^. Sava et al.^60^ experimented with Faster R-CNN and YOLO models for detecting the brown marmorated stink bug (i.e., *Halyomorpha halys*) in tree images. Experimental results on a dataset of images assembled from the the Maryland Biodiversity Project^61^ showed that the YOLOv5m variant produced the best results, with an *mAP* of 99.2%, as compared to the Faster R-CNN which had an *mAP* of 89.1%. In contrast to that, Takimoto et al.^54^ showed that Faster R-CNN was better than YOLOv4 for detecting herbivorous beetles, specifically, striped flea beetle (i.e., *Phyllotreta striolata*) and the turnip flea beetle (i.e., *Phyllotreta atra*) in a set of images collected from the web and through fieldwork. Similarly, Ozdemir and Kunduraci^57^ also found the Faster R-CNN network (with Inception-v3^62^ backbone) to be better than YOLOv4 when used to detect and classify insects according to order level (using a dataset consisting of 25,820 training images and 1,500 test images). Butera et al.^63^ also showed that Faster R-CNN (with MobileNet-v3^48^ backbone) represents an effective model for detecting beetle-type pests (specifically, *Popillia japonica*) and also for distinguishing them from other types of non-harmful but similar looking beetles (*Cetonia aurata* and *Phyllopertha horticola*), giving an overall *mAP* of 92.66%. The dataset used contained 36,000 images collected from the web and photo sharing sites. Ahmad et al.^64^ also used the web to assemble a dataset of 7,046 images which contain 23 types of pests. They experimented with a set of YOLO models and showed that YOLOv5-X gave the best results overall, with an *mAP*@0.5 value of 98.3%, and an *mAP*@.50 : 0.05 : .95 value of 79.8%.

In addition to work on deep learning for automated pest identification, recent studies have also focused on identification of beneficial insects such as pollinators and natural predators^65–69^, including Coccinellidae beetles^70,71^. Ratnayake et al.^66^ used a hybrid approach that combines an object detection model (specifically, YOLOv2^23^) with a background subtraction technique to identify and track honeybees in wildflower clusters. The proposed approach (called HyDaT), which can track one insect at a time, was tested on a dataset consisting of 22,260 video frames (with 17,544 bees visible) and it had a detection rate of 86.6%, as compared to a detection rate of 60.7% for YOLOv2. Ratnayake et al.^72^ extended the HyDaT approach^66^ to make it is suitable for tracking multiple insects simultaneously. Their proposed approach (called Polytrack) uses YOLOv4 together with both foreground and background segmentation to identify and track honeybees. Experimental results on 39,909 video frames, including 5,291 frames with honeybees, showed that Polytrack achieved values of 0.975 and 0.972 for precision and recall, respectively, being superior to both HyDaT and YOLOv4 models used by themselves. Bjerge et al.^69^ assembled a dataset consisting of 29,960 beneficial insects in nine taxa (such as bees, hoverflies, butterflies and beetles) and used the dataset to study the usability of YOLO models to accurately detect and classify such insects. Experimental results showed that the YOLOv5 model had the best performance with an *mAP*@0.50 : 0.05 : 0.95 of 0.592, and a best F1-score of 0.932. Similarly, Spanier^68^ assembled a dataset of approximately 17,000 imaged of pollinator insects of eight types (including bees and wasps, butterflies and moths, beetles, etc.) retrieved from the iNaturalist (inaturalist.org) and Observation.org databases. The best performing model, a variant of YOLOv5, achieved an overall accuracy of 0.9294 and F1-score of 0.9294. Bjerge et al.^59^ constructed a dataset of 100,000 annotated images containing small insects. The authors experimented with Faster R-CNN models and YOLOv5 models. To enhance the detection, they proposed a motion-informed-enhancement of the images. Experimental results showed that YOLOv5 achieved an *mAP*@0.50 value of 0.924, while the Faster R-CNN model achieved an *mAP*@0.50 value of 0.900.

In terms of coccinellid beetle detection, Venegas et al.^71^ used traditional image processing techniques (based on saliency maps, linear iterative clustering and active contour) to identify RoIs (bounding boxes) that can potentially contain coccinellids, and subsequently used a deep CNN to classify the RoIs as coccinellids or not-coccinellids. The approach was evaluated on a dataset of 2,300 coccinellid beetle images assembled from the iNaturalist project in Ecuador and Colombia. The RoI detection approach had an accuracy of 92%, while the CNN model had an area under the curve (AUC) of 0.977. Similarly, Vega et al.^70^ used a CNN together with the weighted Hausdorff distance as a loss function to detect beetles in a dataset of 2,633 images similar to the ones used by Venegas et al.^71^, and reported a mean accuracy of 94.30%. While these works represent important first steps towards automated identification of coccinellid beetles (considered to be natural pest controllers), the realm of deep learning for object detection to automatically detect and classify coccinellids found in sorghum is largely unexplored.

The conventional manual identification of coccinellids requires expert skills and identification keys based on coloration and morphological characteristics. In contrast, existing automated tools based on digital technologies and imagery data do not employ state-of-the-art deep learning architectures and may not be very accurate^73^. Thus, a vision-based automated system for image processing using deep neural networks needs to be researched for precise classification and identification of coccinellids to advance the integrated pest management area in sorghum. Towards this goal, we first assembled a dataset consisting of approximately 5,000 images retrieved from iNaturalist. The dataset assembled was used to study automated deep learning approaches to enable the detection and classification of coccinellids. We trained variants of the popular two-stage Faster R-CNN model, enhanced with FPN, a model referred to as Faster R-CNN-FPN. We also trained variants of the YOLOv5 and YOLOv7 models. We choose to focus on the Faster R-CNN-FPN model, given that this model has shown best performance in some prior related works^40,54,57^. As backbone CNN, we explored ResNet-50 and ResNet-101 given that these networks commonly lead to a good trade-off between accuracy and speed^74^. Similarly, we selected YOLOv5 as another strong model to experiment with given its best performance in several prior works^59,64,69^. Finally, we also choose to include YOLOv7 in our study, as it gives best performance on several benchmark datasets^28^ and it has not been explored for insect detection (neither pests nor beneficial insects) in the IPM area. To summarize, our research contributes a dataset and effective deep learning models trained to detect and classify coccinellids, including Faster R-CNN-FPN, YOLOv5 and YOLOv7 models. To the best of our knowledge, this is the first study to explore YOLOv7 for insect detection and classification. Our best models can potentially be installed and used on unmanned vehicles to automate the detection and classification of coccinellids in sorghum fields during field scouting. The models can be further customized to other natural enemies encountered in different crops during automated field scouting.

## Methods

### Deep learning approaches for object detection

**Figure 1:**
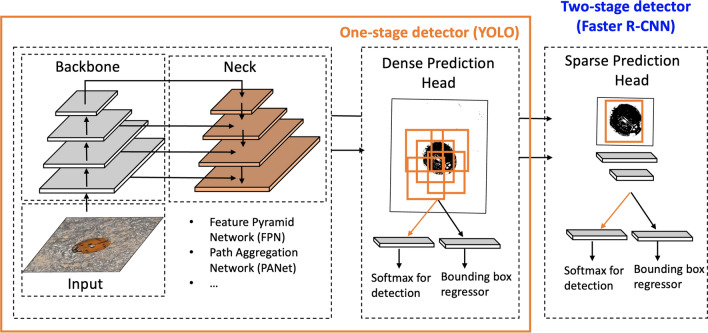
Generic architecture for object detection approaches. A modern object detection network consists of three main components: (1) a *backbone* network that performs feature extraction for a given *input* image; (2) a *neck* that collects and combines features from different layers; and (3) a *head* which is used to detect and classify objects of interest. One-stage detectors use a dense prediction head to simultaneously address the detection (bounding box regression) and classification tasks, while two-stage detectors decouple the two tasks and use a sparse prediction head to classify previously identified RoIs.

The generic architecture of deep neural networks for object detection consists of two main components: a *backbone*, which is commonly a pre-trained CNN network used to generate feature maps, and a *head*, which is used to detect objects as bounding boxes defined by their coordinates (bounding box prediction) and to classify objects into one of several categories of interest^25^, in our case, different types of coccinellids. One-stage detectors, including the YOLO family of detectors, have a dense prediction head that achieves the object detection and classification tasks simultaneously. Two-stage detectors, including the popular Faster R-CNN detector, decouple the object detection and classification tasks and achieve them in two stages. In the first stage, they use a dense prediction head to generate RoIs that may contain objects. In the second stage, a sparse detection head is used to classify the RoIs according to different object categories and to refine their bounding boxes. In recent years, it has become standard practice to insert a *neck* in between the backbone and the head of the network, to collect and mix features from different layers. The FPN network^31^ is one example of a neck that is commonly used in object detection networks. FPN uses a top-down path with lateral connections to extract semantic feature maps at different scales^25^. The resulting feature maps enable the model to find objects at different scales. Path aggregation network (PANet)^75^ is another example of a neck used in object detectors. It enhances FPN with a bottom-up path which helps propagate the low-level features. Equipped also with an adaptive feature pooling, PANet has been shown to improve object localization^25^. The generic architecture of the one-stage and two-stage detectors is shown in Fig. 1. We study the popular Faster R-CNN as a representative two-stage approach and two YOLO variants, YOLOv5 and YOLOv7, on the task of detecting and classifying common coccinellid found in sorghum. All models studied were trained and evaluated using images annotated with the Labelbox tool (https://labelbox.com).

#### Faster R-CNN-FPN

 Modern Faster R-CNN models use a pre-trained CNN as a backbone for feature extraction combined with an FPN network as a neck to obtain semantic feature maps at different scales. Extracted feature maps are provided as input to a region proposal network (RPN) which can be seen as the dense prediction head of the network. The RPN identifies Regions of Interest or RoIs (i.e., regions that may contain objects of interest - in our case, coccinellids) and their corresponding locations (i.e., rectangular bounding boxes parameterized using the box’s center coordinates, and its height and width). More precisely, the RPN uses a sliding window to generate three anchors with different aspect ratios (1:2, 1:1 and 2:1, respectively) at each grid cell in each input feature map. The anchors are labeled (as *object/positive* or *background/negative*) based on their overlap with ground truth bounding boxes and used to train the RPN network to identify RoIs and their locations. Highly overlapping regions, potentially corresponding to the same object, can be filtered using a non-maximum suppression (NMS) threshold. Subsequently, the resulting RoIs together with the feature maps are provided as input to a sparse prediction head which is trained to classify RoIs into several categories of interest (e.g., different coccinellid types) and refines their locations. All parameters of the network are trained together using a multi-task loss, which combines the cross-entropy classification loss with a linear regression *L*2 loss^76^. We experiment with two CNN networks pre-trained on ImageNet^77^ as the CNN backbone, specifically, ResNet-50 and ResNet-101 networks, given that they provide a good trade-off between accuracy and speed^74^. The FPN produces features maps at 5 different scales, and consequently 5x3 anchors are generated at each location. Rezatofighi et al.^78^ suggested that the standard *L*2 loss used to regress the parameters of the bounding box corresponding to an object is not strongly correlated with the IoU (Intersection over Union) metric generally used to evaluate object detection approaches. Instead of the *L*2 loss, they proposed to use a loss based on the IoU metric. Specifically, they experimented with a IoU loss and a loss based on a generalized IoU (GIoU), and showed that optimizing the GIoU loss helps improve the performance measured either using the GIoU itself or the standard IoU. Given this result, we experiment with the IoU and GIoU as the regression loss for the bounding box regression task in Faster R-CNN.

#### YOLOv5

As described above, two-stage detectors, such as Faster R-CNN-FPN network, re-purpose image classification to perform object detection by using the RPN to identify anchors that contain objects of interest as RoIs, and subsequently classifying the RoIs into specific categories. As opposed to that, one-stage detectors use directly the input image and to identify bounding box coordinates and class probabilities for objects of interest. YOLOv5 was released in 2020 by a company called Ultralytics^26^ and has evolved over time. We used the latest YOLOv5 (v6.0/v6.1) architecture^79^. The backbone for YOLOv5 is a New CSP-Darknet53 which combines the original Darknet53 network used in YOLOv3^24^ with the CSPNet network^80^. Darknet53 was inspired by the ResNet architecture and it was specifically designed for object detection. CSPNet addresses the issue of duplicate gradient information in large backbone networks by truncating the gradient flow to speed up computation. The current neck used in the YOLOv5 architecture consists of two components, SPPF and New CSP-PAN. SPPF is a variant of the Spatial Pyramid Pooling (SPP)^81^, which helps identify small objects and also objects at different scales. SPPF was designed to improve the computation speed of SPP. Similar to the Darknet53 backbone, the PAN network (PANet)^75^ is also combined with CSP to improve computation speed. YOLOv5 uses a dense prediction head which is inherited from YOLOv3^24^.

In addition to components that improve efficiency, YOLOv5 makes use of a variety of augmentation techniques on the input image. Among others, mosaic augmentation^25^ is used to stitch together four images with the goal of training the model to find objects in places other than the center of the image, where a large majority of objects are generally located. Furthermore, YOLOv5 uses automatically generated anchors (with different scales and aspect ratios) to predict bounding boxes (and confidence scores) for each cell in a grid directly from the input image. The anchors are generated using k-means clustering based on the bounding boxes in the training set^23^ and a genetic evolution algorithm that optimizes the initial k-means centroids based on the complete IoU (CIoU) loss^82^. The CIoU loss aggregates the overlap area, distance between center points, and aspect ratio consistency of two bounding boxes. The YOLOv5 head has 3 detection layers corresponding to three different scales and predicts bounding boxes with 3 different aspect ratios for each scale, resulting in a total of 9 anchors. The bounding boxes are predicted as deviations from the anchor dimensions. As in Faster R-CNN-FPN, the NMS technique is used to filter bounding boxes representing the same object. The whole network is trained using a multi-task loss, which combines classification loss (binary cross-entropy), objectness loss (binary cross-entropy) and location loss (CIoU). YOLOv5 uses an exponential moving average (EMA) of the model checkpoints as final detector. YOLOv5 itself represents a series of object detection models (compound-scaled variants of the same architecture) that have been pre-trained on the MS COCO dataset^49^. Models in the YOLOv5 series have different sizes as applications have different needs in terms of the trade-off between accuracy and speed. In this study, we experiment with five YOLOv5 variants that vary in size from nano (YOLOv5n) to small (YOLOv5s), medium (YOLOv5m), large (YOLOv5l) and extra-large (YOLOv5x), whose specific architectures are available from the official GitHub repository^26^ as .yaml files in the models directory.

#### YOLOv7

 The YOLOv7 architecture has been designed based on the “bag-of-freebies” idea introduced by Bochkovskiy et al.^25^, which refers to the fact that while it’s important for a detector to be fast at inference time, the training can be more expensive if it helps to improve the overall accuracy of the model (this is acceptable as the training is done offline). With this idea in mind, YOLOv7 introduced several innovations in network architecture and training strategies. One important innovation, the main component of YOLOv7’s architecture (used both in the backbone and neck/head networks), is a block called extended efficient layer aggregation network (E-ELAN). ELAN^83^ uses a “stack in computational block” structure combined with CSP to optimize the shortest gradient path and ensure that scaling up the network does not result in deterioration of performance. In addition, E-ELAN uses “expand, shuffle, merge cardinality” which enables the model to learn more diverse features.

The neck of the network is structured based on a PAFPN network (a combination of PAN and FPN) which uses E-ELAN for feature extraction and fusion. In addition to the “lead head”, YOLOv7 introduces an “auxiliary head”, somewhere in the middle of the network, meant to assist the “lead head” (which may be too far down the network). Soft labels are assigned to the auxiliary and lead heads in a coarse-to-fine manner based on the predictions of the lead head and the ground truth. The coarse soft labels used by the auxiliary head represent a relaxed version of the fine soft labels used by the lead head, as it is expected that the auxiliary head is less precise. In terms of anchors, YOLOv7 leverages the automated anchor selection approach proposed in YOLOv5^26^ and uses 3 aspect ratios for each of the 3 features maps representing three different scales (for a total of 9 anchors). Furthermore, data augmentation techniques similar to those used in YOLOv5 are also used in YOLOv7 (including mosaic augmentation), and the commonly-used NMS technique is employed to filter out the predicted bounding boxes. To achieve robustness through module-level re-parameterization (i.e., aggregating the weights of a multi-branch module during inference), YOLOv7 uses gradient flow propagation paths to “plan” what modules can benefit from re-parameterization.

YOLOv7 is trained from scratch on the COCO dataset using a multi-task learning loss consisting of the classification loss (binary cross-entropy), objectness loss (binary cross-entropy) and location loss (CIoU). If both the lead and the auxiliary heads are used, the loss includes similar components corresponding to the two heads, with different weights. The final model used for inference is based on an EMA of the model parameters at different checkpoints during training. Together, the innovations introduced in YOLOv7 and the components reused from prior works have led to state-of-the-art results on standard benchmark datasets for object detection^28^, and at the same time, smaller inference time as compared with other YOLO models. YOLOv7 also consist of a series of pre-trained models of various sizes. In this study, we experiment with four YOLOv7 variants: 1) the standard YOLOv7 model designed for standard GPUs and 2) its compound scaling variant, YOLOv7-x; 3) the smallest model, YOLOv7-tiny deigned for edge GPU; and 4) a larger model, YOLOv7-d6, a cloud GPU architecture.

### Dataset

**Figure 2:**
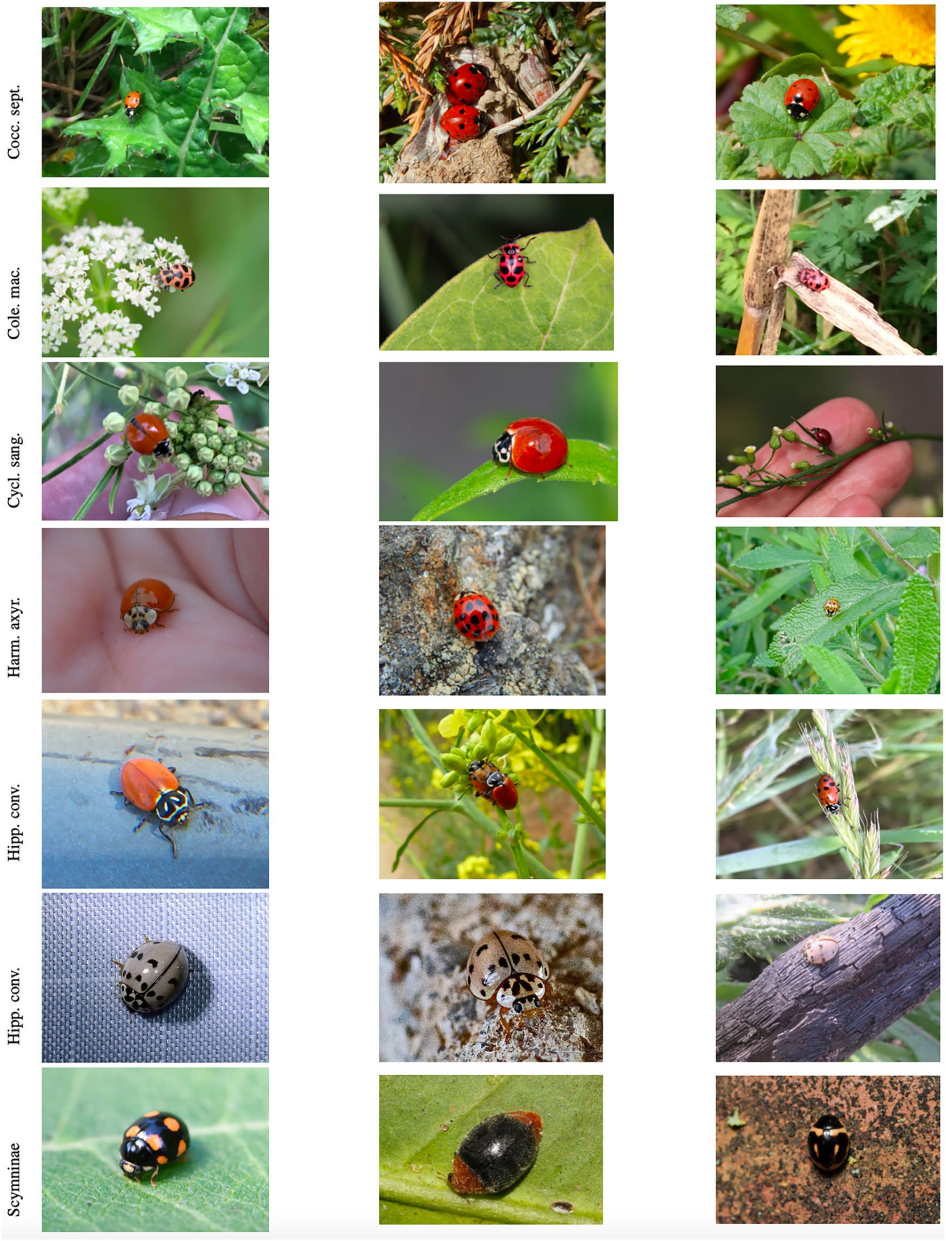
Samples of 3 coccinellid images in each of the 7 categories included in the study.

Coccinellid imagery downloaded from the iNaturalist web portal was used (inaturalist.org). iNaturalist is a citizen science project that allows naturalists to upload and share observations (i.e., images) of biodiversity worldwide through a web platform and mobile app for free. Submission by observers include the actual images, their locations, observed time, and group identifications. Agreements on the taxa in the observations create a “research-grade” label that is assigned to the observation. iNaturalist makes an archive of research-grade observation data available to the environmental science community via the Global Biodiversity Information Facility (GBIF)^84^. We used GBIF to assemble a dataset for training and testing deep learning models for the detection, localization and classification of coccinellids. Only research-grade labels at family, genus, and species level were considered in the dataset that we assembled. The dataset includes seven distinct categories of coccinellids corresponding to the most important coccinellids found in sorghum plants, specifically: *Coccinella septempunctata*, *Coleomegilla maculata*, *Cycloneda sanguinea*, *Harmonia axyridis*, *Hippodamia convergens*, *Olla v-nigrum* and the subfamily Scymninae^17^. Three sample images in each of these seven categories are shown in Fig. 2 (where each row corresponds to one coccinellid type).

We aimed to select approximately 700 images per category, and assembled a dataset with a total number of 4,865 images. Each image contains one or more instances of coccinellids of the same type (i.e., corresponding to a particular category). The dataset was split into train (3,053 images), development or dev (1,113) and test (699) subsets. Table 1 shows the distribution of the images/coccinellid instances over the seven categories in each of the train/dev/test subsets and also in the whole dataset. Supplementary Table S1 shows the distribution of the images with respect to the number of instances per image (1, 2, 3, 4, 5, 6, 7, or 8) for each category and for each of the the train/dev/test subsets. As can be seen, most images have only one or two coccinellid instances, although there are some images that have up to 8 coccinellid instances.

**Table 1:** Dataset statistics. Distribution of the images and coccinellid instances over the seven categories in each of the train/dev/test subsets and also in the whole dataset.

Data	Category	Cocc. sept.	Col. mac.	Cycl. sang.	Har. axyr.	Hipp. conv.	Olla nigr.	Scym.	Total
Train	Images	439	436	423	443	436	436	440	3053
	Instances	461	485	435	475	482	448	446	3232
Dev	Images	160	164	155	157	153	164	160	1113
	Instances	165	182	160	163	172	167	167	1176
Test	Images	99	100	100	100	100	100	100	699
	Instances	102	101	107	100	119	103	102	734
Total	Images	698	700	678	700	698	700	700	4865
	Instances	728	766	702	738	773	718	715	5142

We also classified the instances in our dataset according to their size, as this information is frequently used when evaluating object detection approaches^85^. Specifically, instances are classified based on the area that they occupy in an image as small ($area \le 32^2$), medium ($32^2 < area\le 96^2$) or large ($area>96^2$)^85^. Supplementary Table S2 shows the distribution of the small, medium and large instances in the train/dev/test subsets and in the whole dataset. As can be seen, the number of small instances is just 5 in the total dataset and they are all included in the training subset. The number of medium instance is 142, with 18 of those instances being in the test subset. The remaining 4995 instances are large and represent the majority in our dataset. Given this observation, our evaluation metrics will be generic (representing mostly the large category) as opposed to being specifically focused on small, medium and large categories, respectively.

### Implementation details

**Figure 3:**
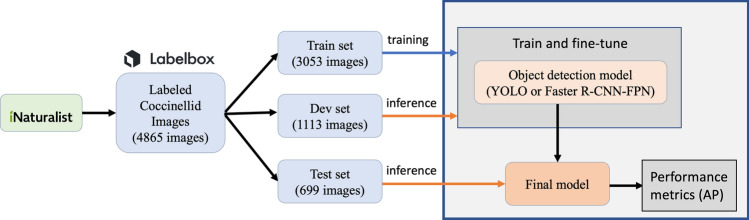
Data flow diagram. Coccinellid images are collected from iNaturalist and labeled using Labelbox. The set of images is split between train, dev and test subsets. YOLO and Faster R-CNN-FPN models trained and fine-tuned on the train and dev subsets, respectively. The performance of each model is estimated on the test subset in terms of average precision (AP) metrics.

We trained and evaluated Faster R-CNN-FPN, YOLOv5 and YOLOv7 models. The data flow diagram for our whole process is depicted in Fig. 3. Training images are used to train the model, while the development images are used to evaluate and select hyperparameters. The performance of the final models is estimated on the test data.

#### Faster R-CNN-FPN

 We used the Detectron2 library for object detection and segmentation^85^ (developed by Facebook Research) to train and evaluate our Faster R-CNN-FPN models for coccinellids detection. Detectron2 uses PyTorch as a deep learning framework and is the successor of Detectron^86^, originally “designed to be flexible in order to support rapid implementation and evaluation of novel research.” The default configuration of the Base-R-CNN-FPN is defined in the “Base-R-CNN-FPN.yaml” file at https://github.com/facebookresearch/detectron2/blob/main/configs/Base-RCNN-FPN.yaml. Hyper-parameters for Detectron2 are available at: https://github.com/facebookresearch/detectron2/blob/main/detectron2/config/defaults.py. The train/test files that we adapted are available https://github.com/cwang16/Detecting-Coccinellids. When training the models, we used the default values for most of the hyper-parameters of Detectron2’s Faster R-CNN-FPN object detection models and experimented with ResNet50/ResNet101 as backbone CNNs and IoU/GIoU as the regression losses, respectively. In terms of number of iterations, we started by training the Faster R-CNN-FPN model with the default 40,000 iterations. However, the learning curves shown in Supplementary Fig. S1

suggested that both training and development losses were still decreasing, while the AP was still increasing after 40,000 iterations. Thus, to enable the network to learn further and give better performance, we ran all experiments for 400,000 iterations, and identified the best iteration for each model based on learning curves as shown in Supplementary Fig. S2. Specifically, the best iteration is identified as the point where the validation loss starts increasing while the training loss is still decreasing. Using this criterion, for Faster R-CNN-FPN with ResNet-50 the best validation/training point was observed around iteration 80,000 for both IoU and GIoU losses, while for Faster R-CNN-FPN with ResNet-101 the best point was observed around iteration 220,000 for both losses.

#### YOLOv5

 We used the official PyTorch YOLOv5 implementation provided by Ultralytics^26^, which is available at https://github.com/ultralytics/yolov5, to train and evaluate the YOLOv5 models. We used the pre-defined model configurations for the YOLOv5 variants used in the experiments, where the number of classes was changed to 7. The pre-defined configurations are available at https://github.com/ultralytics/yolov5/tree/master/models, where the files “yolov5n.yaml”, “yolov5s.yaml”, “yolov5m.yaml”, “yolov5l.yaml”, “yolov5x.yaml”, correspond to the five YOLOv5 variants used in our study, respectively. In terms of hyper-parameters, YOLOv5 provides three different hyper-parameter settings, specifically, “hyp.scratch-low.yaml”, “hyp.scratch-med.yaml” “hyp.scratch-high.yaml”, to train smaller, medium and larger size models, respectively. These three hyper-parameter settings can be found at https://github.com/ultralytics/yolov5/tree/master/data/hyps. When training the models for YOLOv5, we used the default values for most of the hyperparameters, except for using 500 epochs and a batch size of 4. The best model identified by the YOLOv5 framework using the validation data was used for evaluation. As an example, the specific command line used to train the YOLOv5s model is shown below: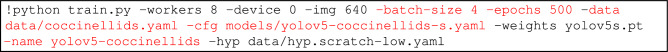 where the modified files and non-default hyper-parameters are highlighted in red font. Similarly, the command lined used to evaluate the YOLOv5s model is:
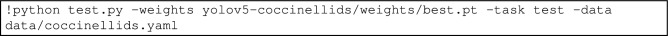


#### YOLOv7

We also used the official YOLOv7 PyTorch implementation^28^ at https://github.com/WongKinYiu/yolov7 to train and evaluate the YOLOv7 models. As for YOLOv5, we used the pre-defined model configurations for the YOLOv7 variants used in the experiments, where the number of classes was changed to 7. The pre-defined configurations are available at https://github.com/WongKinYiu/yolov7/tree/main/cfg/training, where the files “yolov7.yaml”, “yolov7-tiny.yaml”, “yolov7x.yaml”, “yolov7-d6.yaml”, correspond to the four YOLOv7 variants used in our study, respectively. In terms of hyper-parameters, YOLOv7 provides three different hyper-parameter settings for: 1) edge GPU architectures (YOLOv7-tiny); 2) standard GPU architectures (YOLOv7 and YOLOv7-x); and 3) cloud GPU architectures (including YOLOv7-d6). These three hyper-parameter settings can be found at https://github.com/WongKinYiu/yolov7/tree/main/data, where “hyp.scratch.tiny.yaml” is the setting for YOLOv7-tiny, “hyp.scratch.p5.yaml” is the setting for YOLOv7 and YOLOv7-x, and “hyp.scratch.p6.yaml” is the setting for YOLOv7-d6.

When training the models for YOLOv7, we used the default values for most of the hyperparameters, except for using 500 epochs and a batch size of 2. The best model identified by the YOLOv7 framework using the validation data was used for evaluation. As an example, the specific command line used to train the YOLOv7 model is shown below:
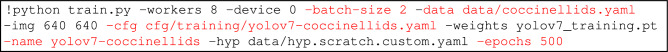
where the modified files and non-default hyper-parameters are highlighted in red font. Similarly, the command lined used to evaluate the YOLOv7 model is:
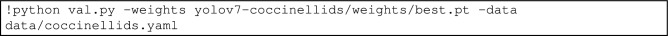


To ensure reproducibility, we make available our train/test/dev data (in the form of iNaturalist image IDs), annotation, model configurations, and best trained models at https://github.com/cwang16/Detecting-Coccinellids.

All the models were trained on Amazon Web Services (AWS) p2.xlarge instances. According to AWS, the configuration of the p2.xlarge instance is as follows: 1 GPU, 4 vCPUs, 61 GiB of memory, and high network bandwidth. Training of the models for the specified number of iterations/epochs took between 8 and 14 days.

### Evaluation metrics

We used three standard average precision metrics^87^ to evaluate the results of our models. The three metrics are defined using the Intersection-over-Union (IoU) measure, which captures the overlap between the predicted bounding box of an instance and the ground truth bounding box of that instance. Specifically, the IoU is defined as the area of overlap (i.e., intersection) divided by the area represented by the union. The metrics used to evaluate the ability of the models to correctly identify the type of coccinellid are: 1) average precision at $IoU=0.50$, denoted by *AP*@0.50; 2) average precision at $IoU=0.75$, denoted by *AP*@0.75; and 3) average precision at $IoU=.50:.05:.95$, which represents the average precision across ten *IoU* thresholds varying from 0.5 to 0.95 with a step size of 0.05, denoted by *AP*@.50 : 0.05 : .95 or simply *AP*. *AP*@*n* considers a prediction to be correct if the *IoU* between the detected instance and the ground truth instance annotation is greater or equal than *n*. For example, *AP*@0.50 considers a prediction to be correct correct if the corresponding *IoU* is greater or equal to 0.50.

In addition to comparing the models in terms of average precision metrics, we also compared the models in terms of number of layers that the specific model architecture used includes, number of parameters of the network, size of the model (MB) and inference time per image (ms). These characteristics can be used to identify small models that are accurate and fast and can be embedded in mobile devices for automated field scouting.

### Ethics statement

The results presented are based solely on experiments with image data. The experiments do not involve live vertebrates and/or higher invertebrates. All experiments were carried out in accordance with relevant guidelines and regulations.

## Results and discussion

The results of the models used in this study, Faster R-CNN-FPN, YOLOv5 and YOLOv7 on the whole test subset are shown in Table 2. Specifically, for each family of models, we report *AP*, *AP*@0.50 and *AP*@0.75 for the model variants considered. The best result in terms of *AP* (with averages values obtained for a range of 10 IoU thresholds from 0.50 to 0.95 with a step of 0.05) is 74.605 and is obtained with the YOLOv7 model. The best *AP* value for a YOLOv5 model is 73.3 and is obtained with the YOLOv5x variant, while the best value for a Faster R-CNN-FPN model is 65.6 and is obtained with Faster-R101-GIoU. These are all significant results given that the best *AP* value for the popular object detection benchmark Microsoft COCO dataset^49^ (which considers *AP* to be its primary metric) is currently 65.4 (as of February 4th, 2023^88^). In terms of *AP*@0.50 (the primary metric for the PASCAL VOC benchmark dataset^89^), the best value is 97.3 and is also obtained using the YOLOv7 model, while the best *AP*@0.50 value obtained with a YOLOv5 variant (specifically, YOLOv5m) is 96.0, and the best value obtained with a Faster R-CNN-FPN variant (Faster-R50-GIoU) is 94.3. Finally, when using the stricter *AP*@0.75, which requires at least $0.75\%$ IoU overlap when comparing the predicted bounding box with the ground truth bounding box of an object instance, the best result is 82.6 and it is obtained with the YOLOv7 model as well, with the YOLOv5x variant following closely. These results are very competitive in terms of numbers reported in the literature for similar problems and show that the models considered in this study, and in particular the YOLOv7 models, have the ability to detect and localize coccinellids in real world images posted on iNaturalist. Overall, on our test data, the standard YOLOv7 model has the best performance among the models in the YOLOv7 family, followed closely by the YOLOv5x model in the YOLOv5 family. Both these models are significantly better than the best Faster R-CNN-FPN model (specifically, Faster-R101-GIoU).

To evaluate the performance of the models in relation to the their size and inference time, Table 2 also shows the number of layers, number of parameters, inference time (ms) and size (MB) for each model. While the Faster R-CNN-FPN models have an average number of parameters (42 and 60 millions with ResNet50 and ResNet101, respectively) as compared to the other models, they have relatively large sizes (165.8 MB and 242.1 MB, for ResNet50 and ResNet101, respectively) and high inference time (approximately, 130 to 140 ms per image). Thus, despite the fact that these are two-stage detectors, with large sizes and high inference times, the Faster R-CNN-FPN models are not very accurate by comparison with the YOLOv5 and YOLOv7 models on the coccinellid images. The YOLOv5 models have the smallest size and inference time overall, but it can be seen that the performance increases with the size of the model, with YOLOv5x (the largest model) having the best performance at an inference time of 28 ms per image. The size of the YOLOv7 variants is overall larger than the size of the YOLOv5 variants. However, the best YOLOv7 model, the standard YOLOv7, has a size of 74.8 MB and an inference time of 19.2 ms per image, which is lower than the inference time of the best YOLOv5x model. It is also worth noting that the standard YOLOv7 model has a large number of layers (specifically, 314 layes) and accordingly a large number of parameters (comparable to the number of parameters in Faster R-CNN-FPN with ResNet50), but it still has very fast inference time, a proof that the “bag-of-freebies” idea used in YOLOv7 gives the intended results.

**Table 2:** Faster R-CNN-FPN, YOLOv5 and YOLOv7 results. The networks are evaluated on the test subset using the best model according to the development subset. The results are reported in terms of *AP*, *AP*@0.50, *AP*@0.75. The Faster R-CNN-FPN (Faster) models are using ResNet-50 (R50) and ResNet-101 (R101) as base models, with IoU and GIoU as the loss, respectively. The best results for models within a family are highlighted with bold, while the best result overall is also shown in italic. For each model, the number of layers, number of parameters, inference time and size are also shown.

Model	*AP*	*AP*@0.50	*AP*@0.75	Number layers	Number parameters	Inference time (ms)	Size (MB)
Faster-R50-IoU	62.9	94.1	74.2	50	42,000,000	130.2	165.8
Faster-R101-IoU	64.7	93.4	74.5	101	60,000,000	141.6	242.1
Faster-R50-GIoU	63.5	**94.3**	73.9	50	42,000,000	127.4	165.8
Faster-R101-GIoU	**65.6**	93.7	**75.6**	101	60,000,000	138.5	242.1
YOLOv5n	67.6	93.1	79.9	157	1,768,636	4.8	3.8
YOLOv5s	70.8	94.5	83.2	191	7,468,160	3.3	14.4
YOLOv5m	73.0	**96.0**	85.1	212	20,877,180	11.6	44.2
YOLOv5l	73.2	95.3	84.7	267	46,140,588	17.7	92.8
YOLOv5x	**73.8**	95.9	**85.6**	322	86,213,788	28.0	173.1
YOLOv7	*74.6*	*97.3*	*86.2*	314	36,514,136	19.2	74.8
YOLOv7-tiny	68.3	94.7	81.1	208	6,023,832	5.7	12.3
YOLOv7-x	68.3	94.1	79.7	362	70,822,872	28.3	142.1
YOLOv7-d6	65.3	90.6	75.2	566	152,967,984	41.8	1200

To gain insights into how the models perform for each type of coccinellids in our dataset, Table 3 shows results in terms of *AP* (averaged over 10 IoU thresholds) for each of the seven types of coccinellids included in the dataset. As can be seen, the standard YOLOv7 model gives the best results for four coccinellid types, specifically *Coccinella septempunctata*, *Coleomegilla maculata*, *Cycloneda sanguinea* and *Hippodamia convergens*, and highly competitive results for the other three types. The YOLOv5x model gives the best results for the *Harmonia axyridis*, *Olla v-nigrum* and the subfamily Scymninae. Thus, based on these results, we can also conclude that the standard YOLOv7 model is a good choice for identifying coccinellids of the types included in our study, closely followed by the YOLOv5x model. The YOLOv7 model performs the best on coccinellids of type *Coccinella septempunctata* with $AP=780.4$, followed by *Coleomegilla maculata*, *Harmonia axyridis* and *Olla v-nigrum* with $AP=76.4$, $AP=76.2$, and $AP=75.5$, respectively. The *AP* of the model is lower for *Cycloneda sanguinea*, subfamily Scymninae and *Hippodamia convergens*, with *AP* values of 72.2, 71.0 and 70.5, respectively.

**Table:** 

	FasterR-CNN\\FPN-R101-GIoU	YOLOv7	YOLOv5x
Max Iteration	400000	400000	400000
learning rate	0.0002	0.01	0.01
momentum	0.9	0.937	0.937
weight_decay	0.0001	0.0005	0.0005
warmup_epochs	1.0	3.0	3.0
warmup_momentum	unused	0.8	0.8
warmup_bias_lr	unused	0.1	0.1
iou_training_threshould	0.3	0.2	0.2
anchors (p3/8)	unused	12,16, 19,36, 40,28	10,13, 16,30, 33,23
anchor(p4/16)	unused	36,75, 76,55, 72,146	30,61, 156,198, 373,326
anchors(p5/32)	unused	142,110, 192,243, 459,401	116,90, 156,198, 373,326

**Figure 4:**
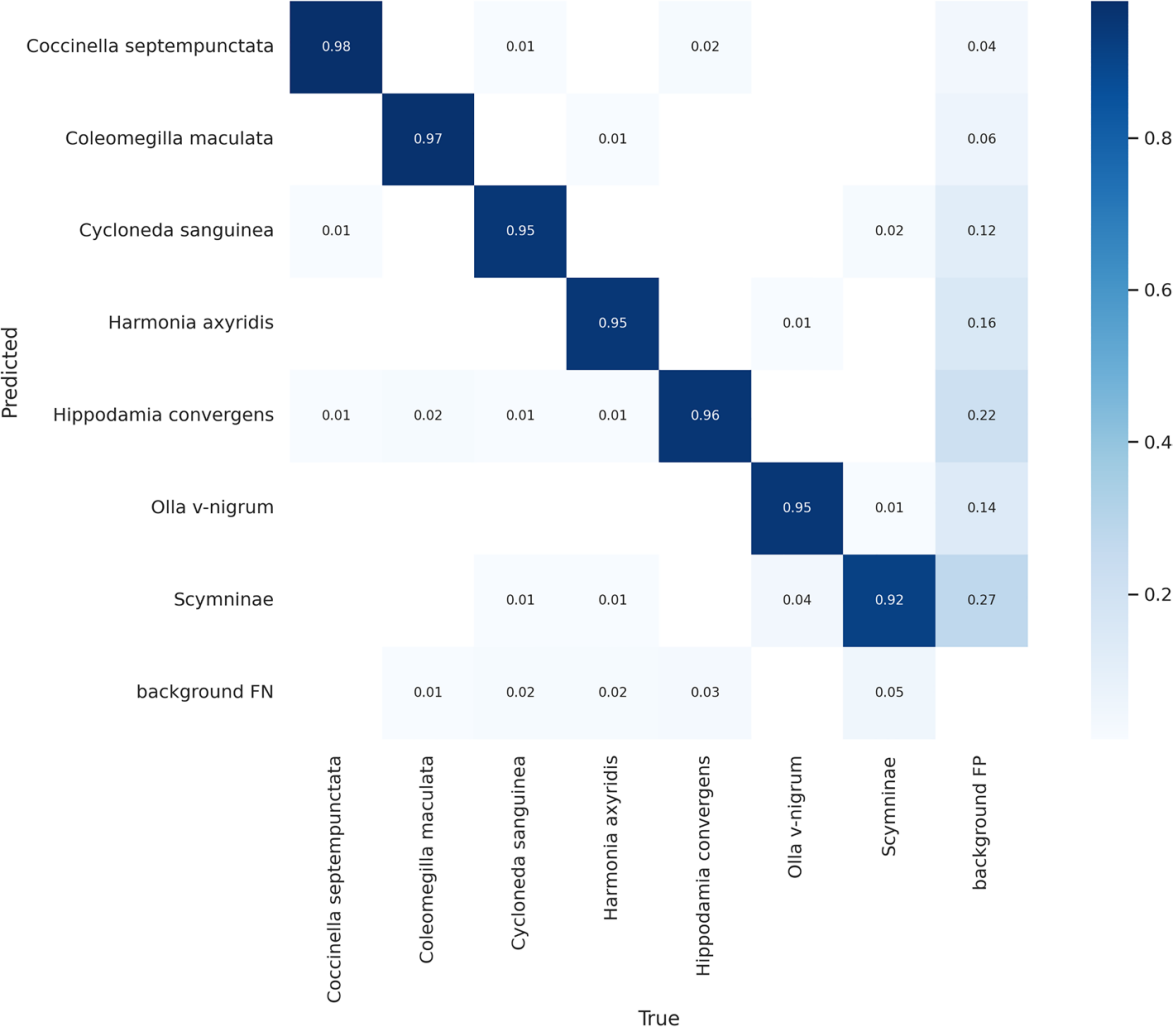
Confusion matrix constructed by comparing the type predicted by YOLOv7 with the manually annotated type. The label *background FN* corresponds to coccinellid instances that were not at all identified by the model, while *background FP* corresponds to predictions of coccinelids when there is not a coccinellid in the original input image.

A confusion matrix constructed by comparing the type predicted by the YOLOv7 model with the manually annotated type is shown in Fig. 4. Generally, the type predicted is correct, with accuracy higher than 92% in all cases. As expected, some detected instances have the type incorrectly predicted. Furthermore, some instances are missed (background FN) or coccinellid instances are detected where there is not a coccinellid in the input image (background FP). Overall, the model makes a small number of false negative mistakes (i.e., it rarely misses a coccinellid), while the largest number of FP mistakes is in the Scymninae subfamily. As can be seen from the confusion matrix, the Scymninae subfamily also has the highest percentage of miss-classified instances overall (8%), while the *Coccinella septempunctata* type has the highest detection accuracy (98%).

**Figure 5:**
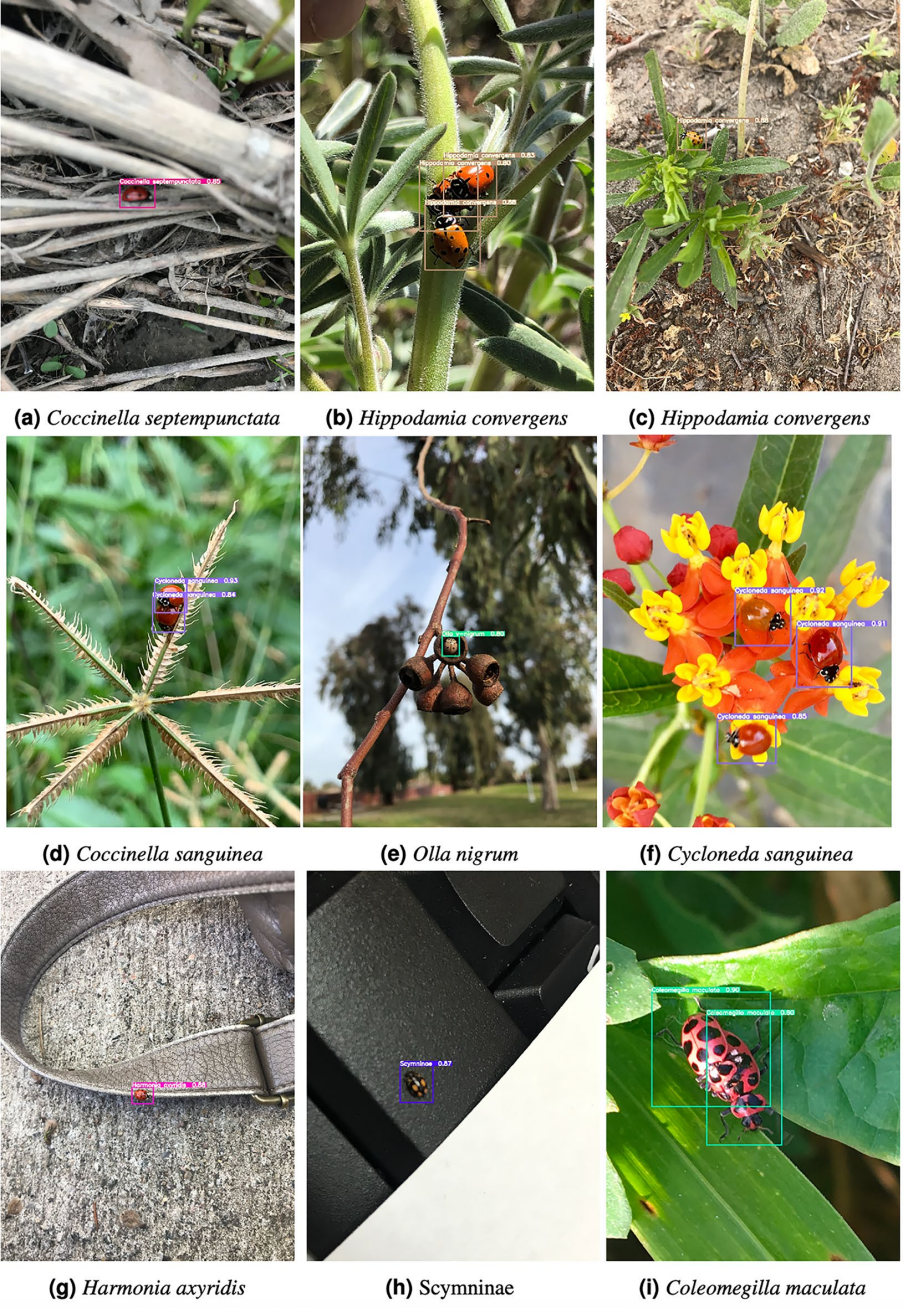
Examples of accurate YOLOv7 predictions on different coccinellid types.

Figure 5 shows examples of correct predictions made for different coccinellid types appearing in somewhat challenging settings. For example, in images **(a)**, **(c)**, **(g)** and **(h)**, the coccinellids are very small, and sometimes hard to see, and they appear in various environments. However, the model correctly identifies them. In images **(b)**, **(d)**, **(f)** and **(i)**, there are multiple coccinellids in one image but the model correctly identifies all of them and their right type, even when they are overlapping. In image **(e)**, there is a coccinellid inside the stem. Supplementary Fig. S3 shows similar examples of correct predictions made by the Faster R-CNN-FPN model with ResMet101 backbone and GIoU loss.

**Table 3:** Faster R-CNN-FPN, YOLOv5 and YOLOv7 results for each coccinellids type in the test subset. Results are reported in terms of *AP* obtained with the best model according to the development subset. The Faster R-CNN-FPN (Faster) models are using ResNet-50 (R50) and ResNet-101 (R101) as base models, with IoU and GIoU as the loss, respectively. The best results for a coccinellid type using models within a family are highlighted with bold, while the best result overall is also shown in italics.

Model	*Coccinella septempunctata*	*Coleomegilla maculata*	*Cycloneda sanguinea*	*Harmonia axyridis*	*Hippodamia convergens*	*Olla v-nigrum*	*Scymninae*
Faster-R50-IoU	67.1	62.7	**60.3**	65.2	55.1	67.0	63.0
Faster-R101-IoU	70.0	63.9	58.8	**70.7**	54.5	**69.8**	65.1
Faster-R50-GIoU	66.2	64.5	59.6	68.0	55.6	67.4	63.2
Faster-R101-GIoU	**70.1**	**66.6**	59.7	68.8	**58.3**	69.0	**67.0**
YOLOv5n	72.1	70.5	64.0	71.0	60.9	68.8	67.2
YOLOv5s	76.3	71.8	68.8	72.7	63.9	73.1	69.3
YOLOv5m	**77.2**	72.8	69.0	73.1	68.4	77.0	72.3
YOLOv5l	77.1	73.3	70.1	74.2	**69.2**	75.3	72.0
YOLOv5x	76.8	**74.9**	**70.3**	*76.8*	69.1	*77.6*	*72.8*
YOLOv7	*80.4*	*76.4*	*72.2*	**76.2**	*70.5*	**75.5**	**71.0**
YOLOv7-tiny	75.5	68.8	63.7	72.7	65.3	70.3	61.4
YOLOv7-x	74.8	69.8	65.4	73.1	62.8	69.4	63.1
YOLOv7-d6	74.2	66.4	61.1	72.4	62.8	69.8	50.4

**Figure 6:**
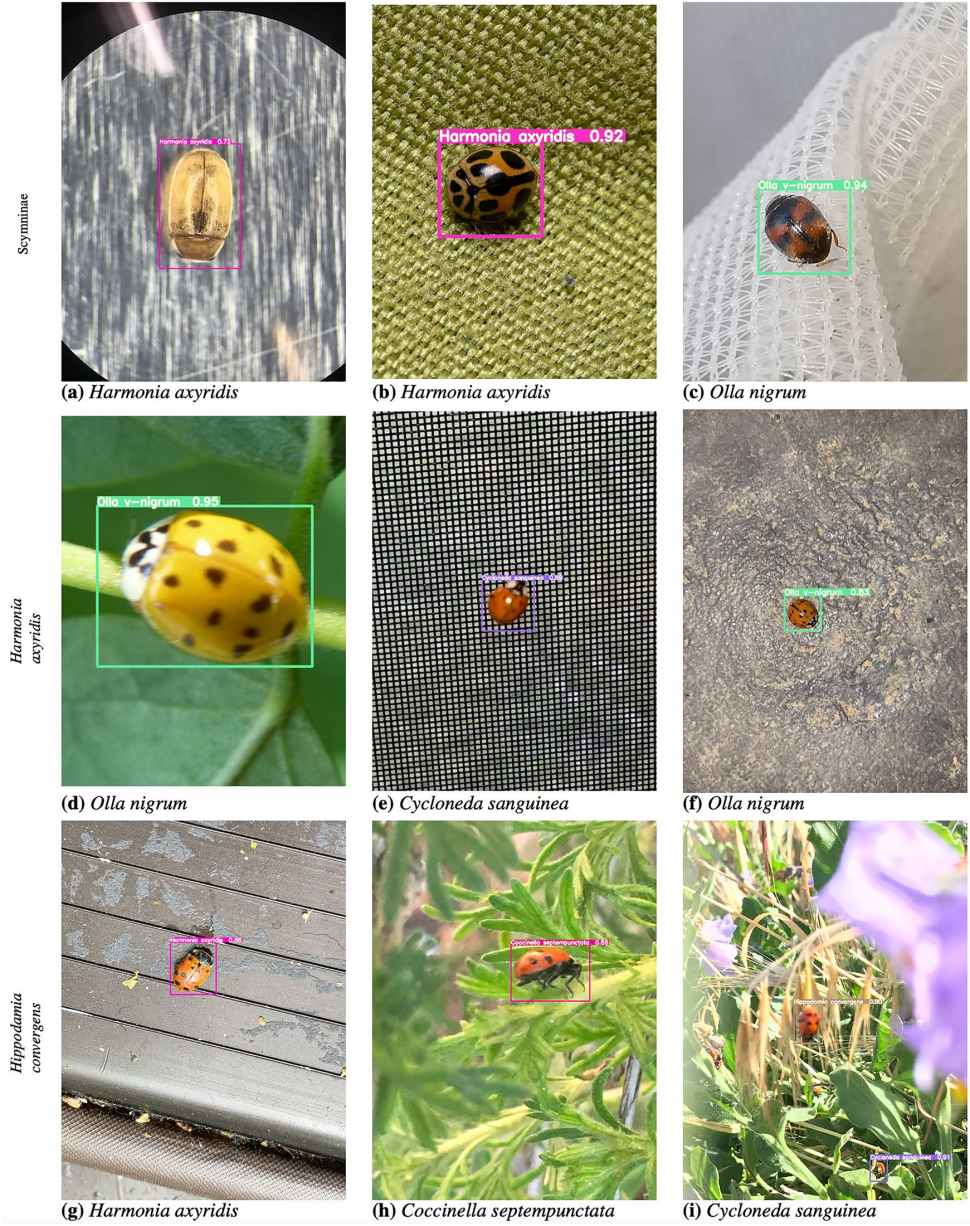
Examples of images where the YOLOv7 model correctly identifies a coccinellids, but the type identified is different from the manually annotated type. Each row shows a ground truth type, specifically, Scymninae, *Harmonia axyridis*, *Hippodamia convergent*. The predicted label for each image is shown below the image.

**Figure 7:**
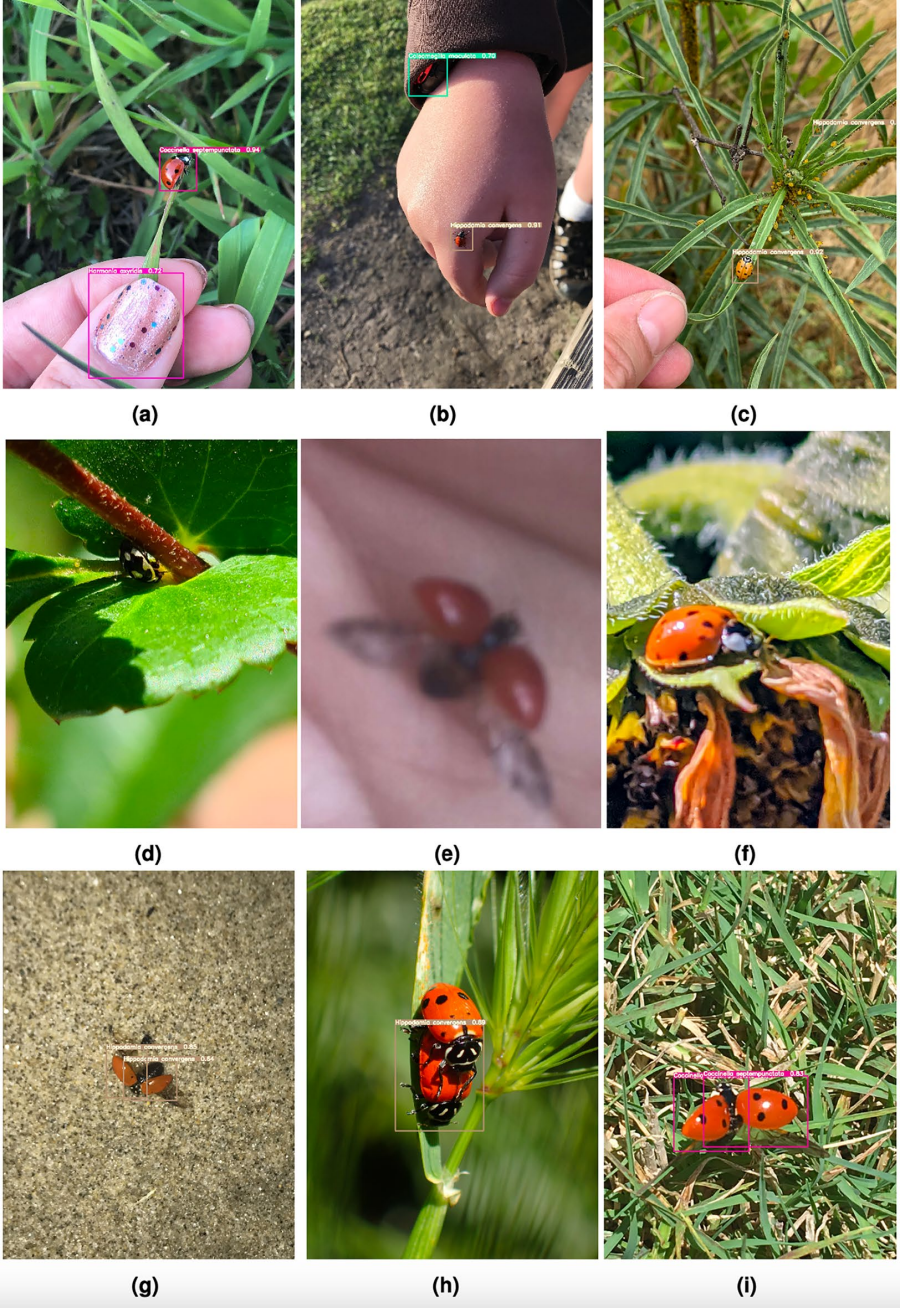
Examples of YOLOv7 errors. Images **(a)**, **(b)** and **(c)** show cases where YOLOv7 is mistakenly detecting an object as a coccinellid. Images **(d)**, **(e)** and **(f)** show cases where YOLOv7 fails to detect a coccinellid. Images **(g)**, **(h)** and **(i)** show cases when YOLOv7 detects two coccinellids instead of one, or alternatively one coccinellid instead of two.

## Error analysis

While the YOLOv7 models work very well, they do have two types of errors: detection errors and localization errors. The detection errors can be grouped into several classes: (1) Errors where a coccinellid is identified but the detected type is wrong, as shown in Fig. 6 (similar errors can be seen for the best Faster R-CNN-FPN model in Supplementary Fig. S4); (2) Errors where a different object in a picture is identified as a coccinellid, as shown in Fig. 7a,–c ; (3) Errors where a coccinellid should be identified but the model completely misses it, as shown in Fig. 7b, e, f; and (4) Errors where a coccinellid has its wings spread out and the model detects two coccinellids instead of one, and conversely, errors where two coccinellids overlap in the image and the model fails to detect two instances, as shown in Fig. 7g, h, and i. It is interesting to see that the YOLOv7 model can identify some coccinellids that overlap as shown in Fig. 5b, d and i but it fails to detect others, as shown in Fig. 7h. When comparing YOLOv7 predictions with Faster R-CNN-RPN predictions, we found that the YOLOv7 model can “fix” some of the errors made by the Faster R-CNN-FPN model, as can be seen in Supplementary Fig. S5.

**Figure 8:**
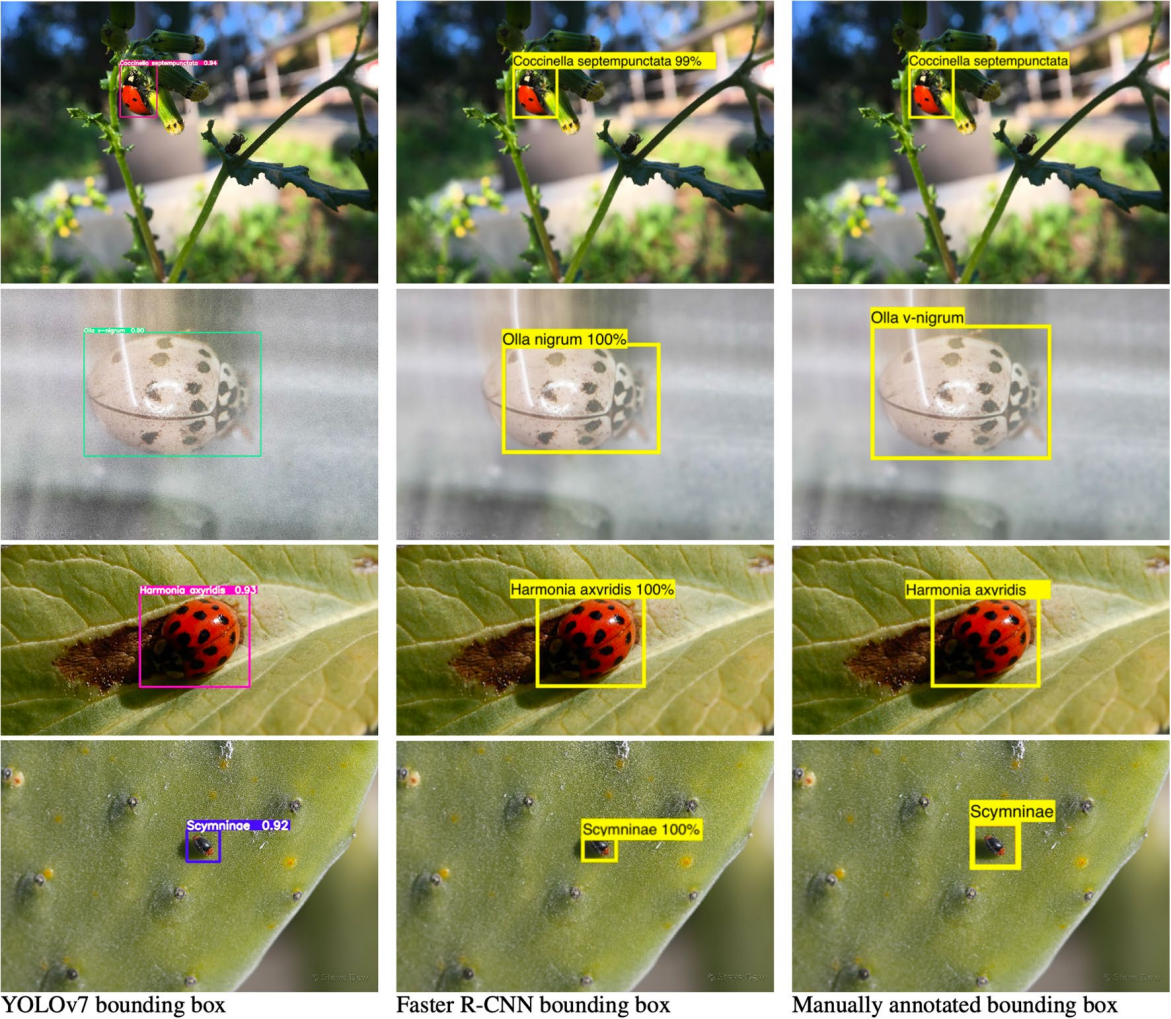
Localization differences. Examples of localization differences between YOLOv7 predicted bounded boxes (first column), Faster R-CNN predicted bounded boxes (second column) and manually annotated bounding boxes (third column).

In addition to detection errors, the models also make localization errors. In particular, a localization error happens when the *IoU* overlap between the predicted bounding box and the manually annotated bounding box does not satisfy the desired threshold (e.g., 50% or 75%). However, to some extent, differences in bounding boxes could stem from differences in the way the manual annotation is performed, as humans are prone to error and inconsistencies when performing annotations, as Fig. 8 shows. Our models learn from the human annotations and will also produce bounding boxes that are enclosing the object of interest more closely or more loosely, thus leading to difference in terms of object localization.

## YOLOv2 model as a tool for coccinellid detection

To enable the use of our best model by the research community, we make available the pre-trained YOLOv7 model as a web-based application at https://coccinellids.cs.ksu.edu, shown in Supplementary Fig. S6. The web-based application is user-friendly and allows the user to explore the model predictions using sample images available on the front page. Alternatively, a new image can be uploaded to the site and submitted for analysis. Underneath, the built-in model is used to detect and classify coccinelids in the image, and the results are displayed on the site using an annotated bounding box. It takes approximately 4-5 seconds for a prediction to be made, given the interaction with the server. In addition to the web-based application, which does not require any programming skills, we will also make the pre-trained models and source code available on GitHub for more experienced users who may need to further train and adapt our models to other species or datasets.

## Limitations of the study

We have shown that deep learning models can be used to identify seven types of common coccinellids in sorghum. While the methodology proposed in this study can provide tremendous benefits to IPM in sorghum, we would also like to point out its limitations:

- As emphasized by our error analysis, some coccinellids are not identified, especially when they cover most of the area of the image, and sometimes when two coccinellids overlap in an image. More images of those types need to be included in the dataset to improve the performance of the models on such images.
- Similarly, the models can make mistakes in terms of the type of the coccinellid identified and also in terms of the precise location (bounding box of the coccinellid).
- While our dataset covers seven types of coccinellids, and includes images with several coccinellids, all coccinellids in an image are of the same type. It is not clear how the models would perform on images with coccinellids of different types.
- Our dataset includes only a small set of images with coccinellids that are considered “small” objects based on their size (see Supplementary Table S2). More data is needed to estimate the performance of the models in detecting “small” coccinellids, which have a high chance to appear in images taken with an autonomous device in the wild.
- While a big variety of images were used to train and test our models, the images may not be fully representative of images that would potentially be taken with an autonomous device such as a drone. Additional data captured with such devices can be added to the training set to improve the robustness of the models in such scenarios.

## Conclusions

In this study, we focus on the application of high throughput deep learning models to detect and classify coccinellids that are commonly found in sorghum. Specifically, we compared two-stage (Faster R-CNN-FPN) and one-stage detectors (YOLOv5 and YOLOv7) on the task of detecting and classifying seven types of coccinellids, including *Coccinella septempunctata*, *Coleomegilla maculata*, *Cycloneda sanguinea*, *Harmonia axyridis*, *Hippodamia convergens*, *Olla v-nigrum*, Scymninae. To do this, we first assembled and annotated a dataset of 4,865 images based on the iNaturalist imagery web server, which publishes citizen’s observations regarding living organisms. Images in the dataset contain between one and eight instances of coccinelids manually annotated with bounding boxes using the LabelBox tool. Using the assembled dataset, split into training, development and test subsets, we experimented with several variants of the Faster R-CNN-FPN network where the base CNN was either ResNet-50 or ResNet-101, and the loss optimized was either the standard IoU or the generalized GIoU. We also experimented with several variants of the YOLOv5 and YOLOv7 models. Experimental results showed that the standard YOLOv7 model gives the best results overall for our test data, with *AP*@0.50 as high as 97.31 and *AP* as high as 74.5 for this specific variant. These competitive results in relation to results for other similar problems in prior works suggest that our models have the potential to make the task of detecting natural enemies in sorghum easier, if integrated in systems for automated pest management. To enable the community to make use of our models, we have made available the models as part of a web-based application that allows end-users to identify coccinellids in their own images.

## Supplementary Information

Supplementary Information.

## Data Availability

The image ids and annotations in the train, dev and test subsets used in this study are made available on GitHub at https://github.com/cwang16/Detecting-Coccinellids.
