# Supplementary material for: Detecting common coccinellids found in sorghum using deep learning models
Source: Sci Rep. 2023 Jun 16;13:9748. doi: 10.1038/s41598-023-36738-5 (PMC10276038; doi:10.1038/s41598-023-36738-5)
Supplement: Supplementary file 1 — Supplementary Information. [file 41598_2023_36738_MOESM1_ESM.pdf]

Detecting Common Coccinellids Found in Sorghum Using Deep Learning Models

Chaoxin Wang[1], Ivan Grijalva[2], Doina Caragea[1,*], and Brian McCornack[2]

[1]Department of Computer Science, Kansas State University, Manhattan, KS 66506, USA
[2]Department of Entomology, Kansas State University, Manhattan, KS 66506, USA
[*]dcaragea@ksu.edu

ABSTRACT

Supplementary Information

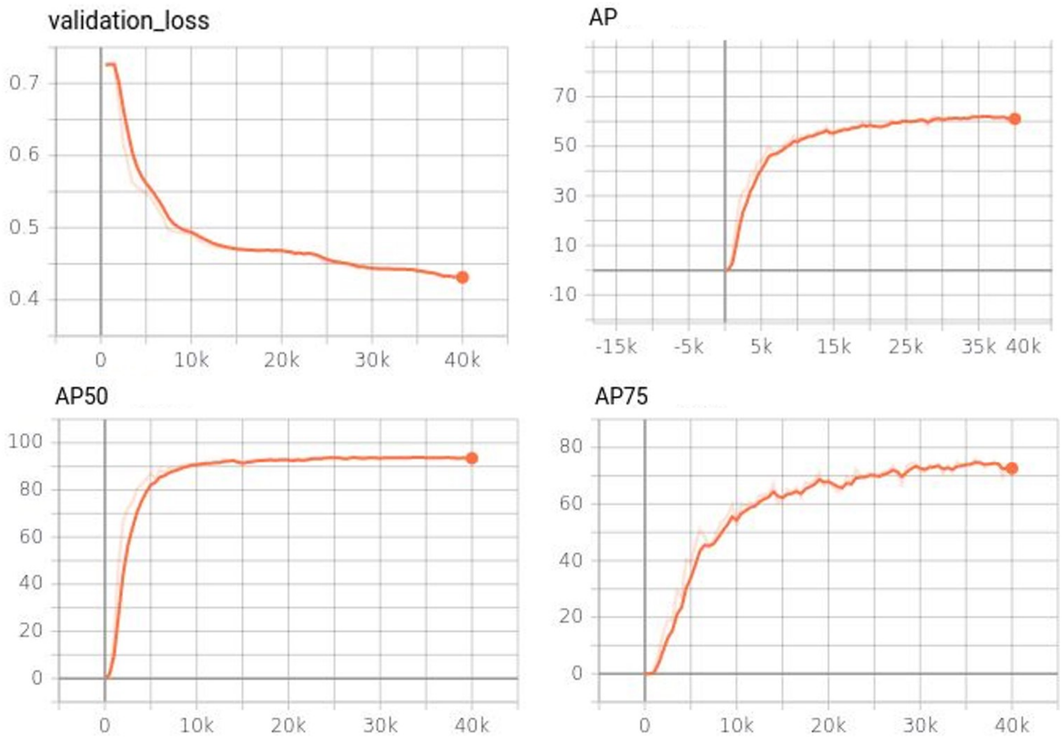

Figure S1. Learning curves of the Faster R-CNN-FPN with ResNet101 and IoU loss on the validation data. Specifically, validation loss, *AP*, *AP*@0.50, and *AP*@0.75 curves are shown when the model is trained for 40,000 iterations. As can be seen, the loss still decreases, while the *AP* curves increase after 40,000 iterations, suggesting the model is still learning.

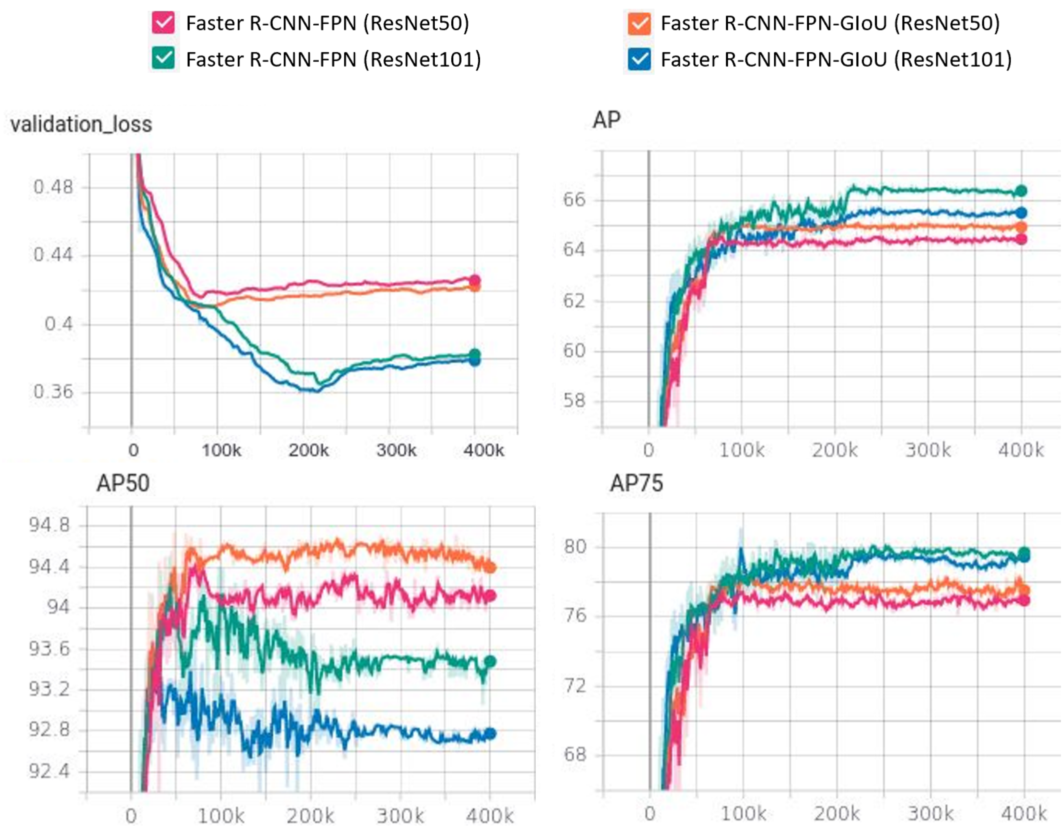

Figure S2. Learning curves of Faster R-CNN-RPN models on the validation data. Specifically, validation loss, AP, $AP@0.50$, and $AP@0.75$ curves are shown when the model is trained for 400,000 iterations.

**Train**	Images per #Instances								Total Images	Total Inst.
Category \ # Instances	1	2	3	4	5	6	7	8		
Coccinella_septempunctata	424	12	2	0	0	0	1	0	439	461
Coleomegilla_maculata	404	26	2	2	0	0	1	1	436	485
Cycloneda_sanguinea	412	10	1	0	0	0	0	0	423	435
Harmonia_axyridis	426	13	0	1	1	1	0	1	443	475
Hippodamia_convergens	397	36	1	1	0	1	0	0	436	482
Olla_nigrum	426	8	2	0	0	0	0	0	436	448
Scymninae	436	2	2	0	0	0	0	0	440	446
**Total**	**2925**	**107**	**10**	**4**	**1**	**2**	**2**	**2**	**3053**	**3232**
**Validation**	Images per #Instances								Total Images	Total Inst.
Category \ # Instances	1	2	3	4	5	6	7	8		
Coccinella_septempunctata	157	1	2	0	0	0	0	0	160	165
Coleomegilla_maculata	150	12	1	0	1	0	0	0	164	182
Cycloneda_sanguinea	150	5	0	0	0	0	0	0	155	160
Harmonia_axyridis	151	6	0	0	0	0	0	0	157	163
Hippodamia_convergens	138	14	0	0	0	1	0	0	153	172
Olla_nigrum	161	3	0	0	0	0	0	0	164	167
Scymninae	155	3	2	0	0	0	0	0	160	167
**Total**	**1062**	**44**	**5**	**0**	**1**	**1**	**0**	**0**	**1113**	**1176**
**Test**	Images per #Instances								Total Images	Total Inst.
Category \ # Instances	1	2	3	4	5	6	7	8		
Coccinella_septempunctata	97	1	1	0	0	0	0	0	99	102
Coleomegilla_maculata	99	1	0	0	0	0	0	0	100	101
Cycloneda_sanguinea	94	5	1	0	0	0	0	0	100	107
Harmonia_axyridis	100	0	0	0	0	0	0	0	100	100
Hippodamia_convergens	90	5	3	0	2	0	0	0	100	119
Olla_nigrum	99	0	0	1	0	0	0	0	100	103
Scymninae	98	2	0	0	0	0	0	0	100	102
**Total**	**677**	**14**	**5**	**1**	**2**	**0**	**0**	**0**	**699**	**734**

Table S1. Distribution of the images with respect to the number of instances per image (1, 2, 3, 4, 5, 6, 7, or 8) for each category and for each of the the train/dev/test subsets.

Size \ Subset	Train	Dev	Test	All
Small	5	0	0	5
Medium	85	39	18	142
Large	3142	1137	716	4995
All	3232	1176	734	5142

Table S2. Distribution of the small, medium and large instances in the train/dev/test subsets and in the whole dataset.

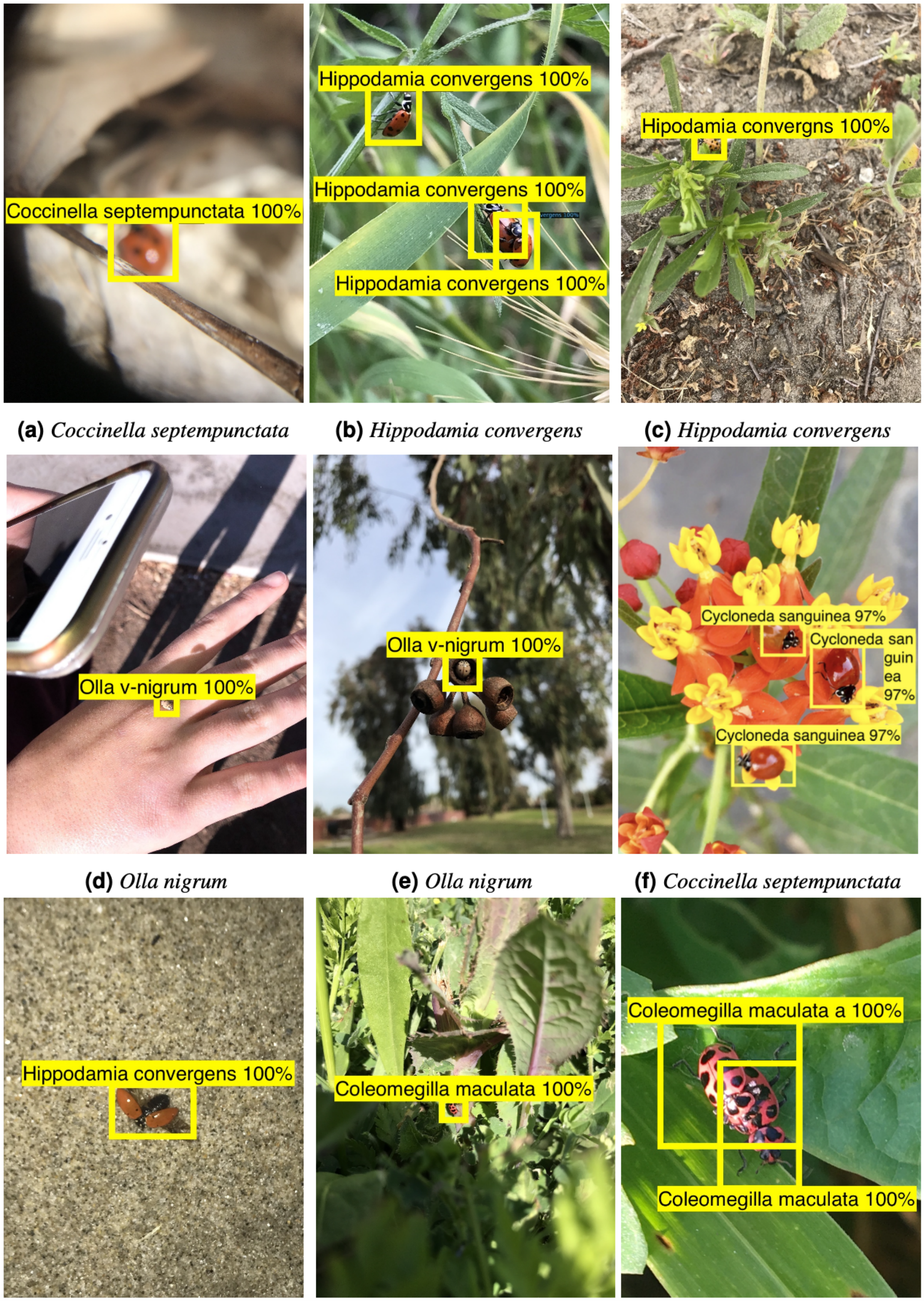

(a) *Coccinella septempunctata* **(b)** *Hippodamia convergens* **(c)** *Hippodamia convergens*

(d) *Olla nigrum* **(e)** *Olla nigrum* **(f)** *Coccinella septempunctata*

(g) *Hippodamia convergens* **(h)** *Coleomegilla maculata* **(i)** *Coleomegilla maculata*

Figure S3. Examples of accurate Faster R-CNN predictions on different coccinellid types

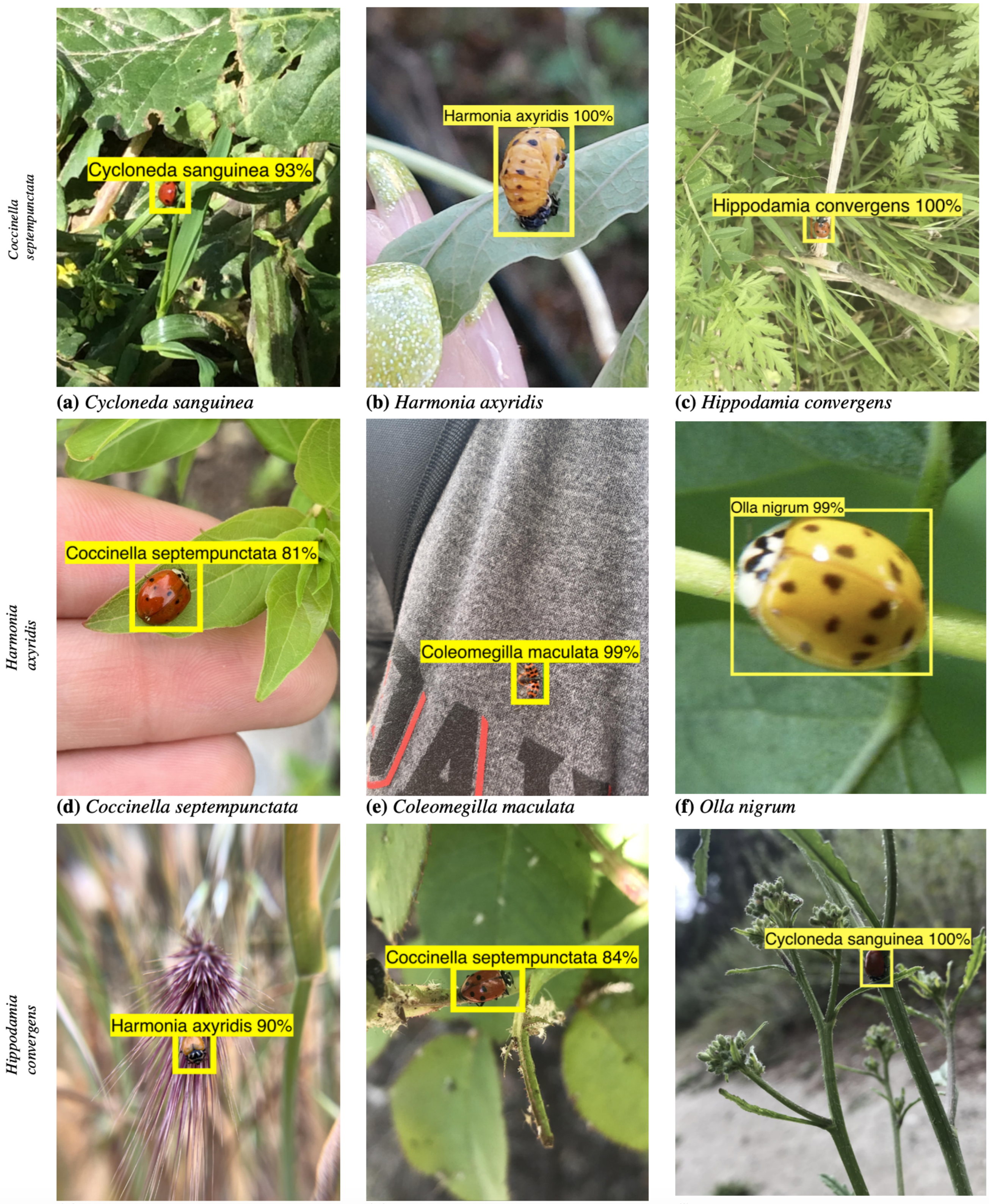

(a) *Cycloneda sanguinea* **(b)** *Harmonia axyridis* **(c)** *Hippodamia convergens*

(d) *Coccinella septempunctata* **(e)** *Coleomegilla maculata* **(f)** *Olla nigrum*

(g) *Harmonia axyridis* **(h)** *Coccinella septempunctata* **(i)** *Cycloneda sanguinea*

Figure S4. Examples of images where the Faster R-CNN model correctly identifies a coccinellids, but the type identified is different from the manually annotated type. Each row shows a ground truth type, specifically, *Coccinella septempunctata*, *Harmonia axyridis*, *Hippodamia convergent*. The predicted label for each image is shown below the image.

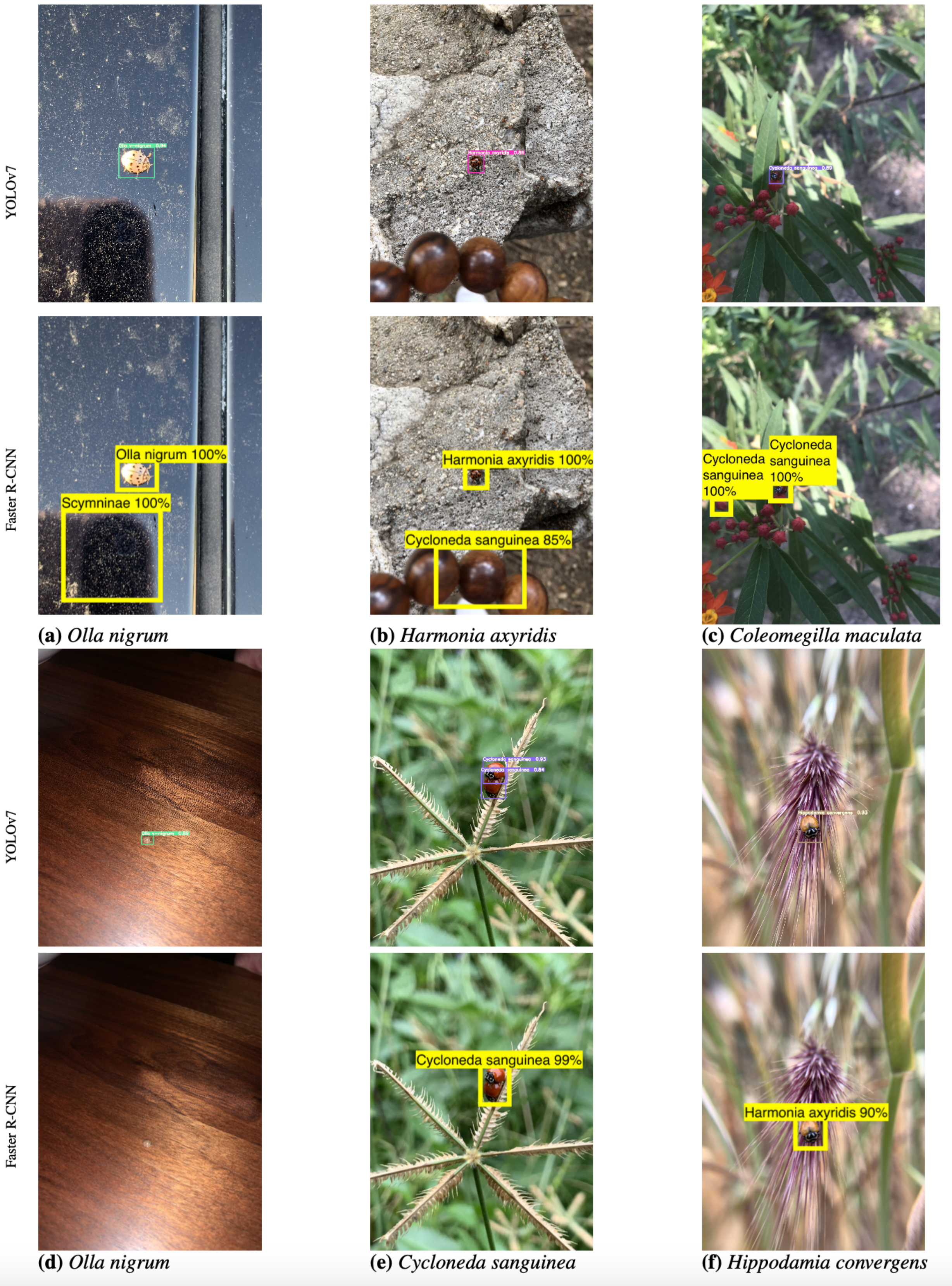

Figure S5. Faster R-CNN errors (bottom) that are fixed with YOLOv7 (top). Examples **(a)**, **(b)** and **(c)** illustrate cases when Faster R-CNN identifies an extra object as a coccinellids in addition to the actual coccinellid that was manually annotated. Example **(d)** shows a case when Faster R-CNN fails to identify the coccinellid in the the image. Example **(e)** shows a case when Faster R-CNN identifies two coccinellids as one. Example **(f)** shows a case when Faster R-CNN identifies a coccinellid but the type if wrong.

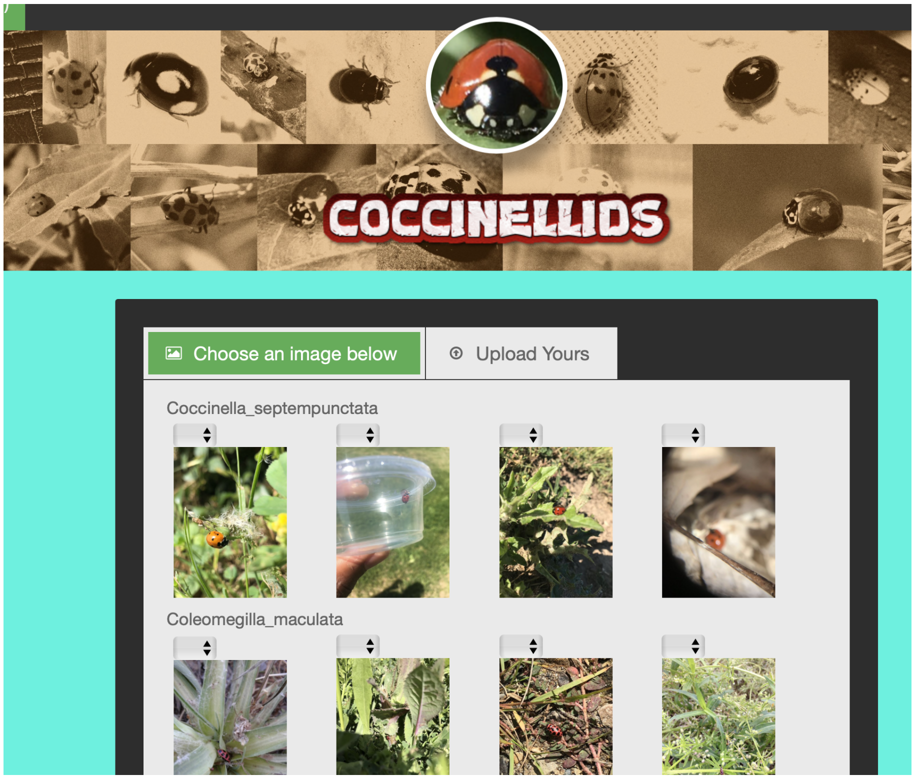

Figure S6. Faster R-CNN-FPN-R101-GIoU model as a web-based application
